# The predictive value of the C-reactive protein-stress hyperglycemia ratio for in-hospital major adverse cardiovascular events in patients with ST-segment elevation myocardial infarction

**DOI:** 10.3389/fcvm.2026.1703043

**Published:** 2026-03-18

**Authors:** Xin Hou, Xiangjie Zhou, Wenli Wang, Xinmin Chen, Yanhui Li, Zheng Gong, Yang Yang, Xiaohong Zhang

**Affiliations:** Department of Cardiology, The Third Affiliated Hospital of Anhui Medical University, Hefei, Anhui, China

**Keywords:** hs-CRP, major adverse cardiovascular events, prognosis, STEMI, stress hyperglycemia ratio

## Abstract

**Background:**

High-sensitivity C-reactive protein (hs-CRP) and stress hyperglycemia ratio (SHR) are recognized as predictors of major adverse cardiovascular events (MACE) in patients with ST-segment elevation myocardial infarction (STEMI). To comprehensively evaluate their combined contribution, we proposed the high-sensitivity C-reactive protein-stress hyperglycemia ratio (CSR). This study aims to assess the predictive value of CSR for in-hospital MACE in STEMI patients.

**Methods:**

This single-center retrospective analysis enrolled patients with STEMI admitted to the Third Affiliated Hospital of Anhui Medical University between October 1, 2021 and June 1, 2025. Based on the incidence of in-hospital major adverse cardiovascular events (MACE), the patients were categorized into MACE and non-MACE groups. Univariate and multivariate logistic regression analyses, restricted cubic spline (RCS) curves, receiver operating characteristic (ROC) curve analysis, and subgroup analyses were conducted to examine the relationship between CSR and in-hospital MACE in STEMI patients.

**Results:**

A total of 246 patients were enrolled, with 56 assigned to the MACE group and 190 to the non-MACE group. The CSR value was significantly higher in the MACE group than in the non-MACE group (*P* < 0.05). Multivariate logistic regression analysis showed that CSR was an independent risk factor for in-hospital MACE in STEMI patients (OR = 1.207, 95% CI: 1.024–1.423, *P* = 0.025). Restricted cubic spline analysis revealed a linear positive correlation between CSR and the risk of in-hospital MACE (P for non-linearity > 0.05). Subgroup analysis indicated that the association between CSR and in-hospital MACE risk was consistent across all subgroups.

**Conclusion:**

CSR is a reliable biomarker for predicting in-hospital MACE in STEMI patients. It may help optimize early risk stratification and potentially improve prognosis in high-risk patients.

## Background

Acute ST-segment elevation myocardial infarction (STEMI) is one of the most critical and devastating types of cardiovascular disease. Although reperfusion therapy and percutaneous coronary intervention (PCI) techniques have continued to advance, the incidence of in-hospital major adverse cardiovascular events (MACE)—such as cardiac death, reinfarction, cardiogenic shock, malignant arrhythmia, and acute heart failure—remains high, significantly impairing short-term prognosis (1, 2). Therefore, identifying effective and early predictive indicators is essential for improving risk assessment and clinical management in these patients.

The pathophysiological process following STEMI involves not only myocardial ischemic necrosis, but also an intense inflammatory response and metabolic stress. C-reactive protein (CRP), an acute-phase protein synthesized by the liver, serves as a sensitive marker of systemic inflammation. Its high-sensitivity form (hs-CRP) can more accurately reflect low-grade inflammatory states (3). Studies have shown that significantly elevated CRP levels in STEMI patients are closely associated with plaque instability, expanded myocardial necrotic area, microvascular dysfunction, and worsened cardiac function, establishing it as an independent predictor of MACE (4–6).

On the other hand, stress hyperglycemia is a common metabolic response during acute myocardial infarction, resulting from sympathetic nervous system activation and the massive release of stress hormones such as cortisol and catecholamines, which lead to insulin resistance and increased hepatic glucose output (7). It is linked to adverse outcomes, including higher mortality and an increased risk of Major Adverse Cardiac and Cerebrovascular Events (MACCE) (8). Admission blood glucose (ABG) has traditionally been used to evaluate this condition; however, ABG can be influenced by factors such as pre-existing diabetes and dietary status. To more specifically quantify acute stress-induced glycemic variability, researchers have proposed the stress hyperglycemia ratio (SHR). By integrating ABG with glycated hemoglobin (HbA1c), which reflects long-term glycemic levels, SHR effectively adjusts for chronic hyperglycemia and provides a purer indication of the severity of acute stress (9, 10). Multiple studies have demonstrated that SHR is a strong predictor of adverse outcomes in AMI patients, showing high predictive value for both short- and long-term prognosis as well as mortality risk (8, 11, 12).

It is noteworthy that inflammation and metabolic stress do not operate in isolation but exhibit profound interactions. Hyperglycemia can exacerbate inflammatory responses by activating oxidative stress and promoting the release of proinflammatory cytokines such as IL-6. Conversely, inflammatory cytokines can further induce insulin resistance and elevated blood glucose, creating a vicious cycle. These mechanisms disrupt neurohormonal homeostasis, potentially impair ischemic preconditioning, enhance a prothrombotic state, and compromise endogenous fibrinolysis, collectively aggravating myocardial injury and worsening prognosis (13–15). Therefore, combined assessment of hs-CRP (representing inflammatory burden) and SHR (reflecting acute metabolic stress intensity) may provide more comprehensive and synergistic predictive information from different pathophysiological dimensions, significantly improving the early identification of high-risk patients. However, most current studies have separately investigated the predictive value of hs-CRP or SHR, with relatively limited research on their combined use for predicting in-hospital MACE in STEMI patients. The synergistic predictive efficacy of these two markers remains to be fully elucidated. To address this, based on previous research experience from Wang Yifei, Chen Jiaying, and others regarding the residual cholesterol inflammatory index (RCII = RC × hs-CRP/10), we proposed a novel index, CSR (hs-CRP × SHR/10), which integrates both markers. Its primary potential utility lies in supplementing or refining these established scores by providing unique, integrated biological information that conventional clinical-composite scores (such as TIMI, GRACE, and Killip) fail to capture. We hypothesize that the combination of CRP and SHR will yield superior predictive performance compared to either marker alone, offering new insights and evidence-based strategies for early risk stratification and optimized intervention in STEMI patients.

## Methods

### Study population

This study was a single-center, retrospective, observational study. It was conducted in accordance with the Declaration of Helsinki and World Health Organization guidelines. The study protocol was approved by the local ethics committee. Written informed consent was waived due to the retrospective and non-invasive nature of the research.

**Figure 1:**
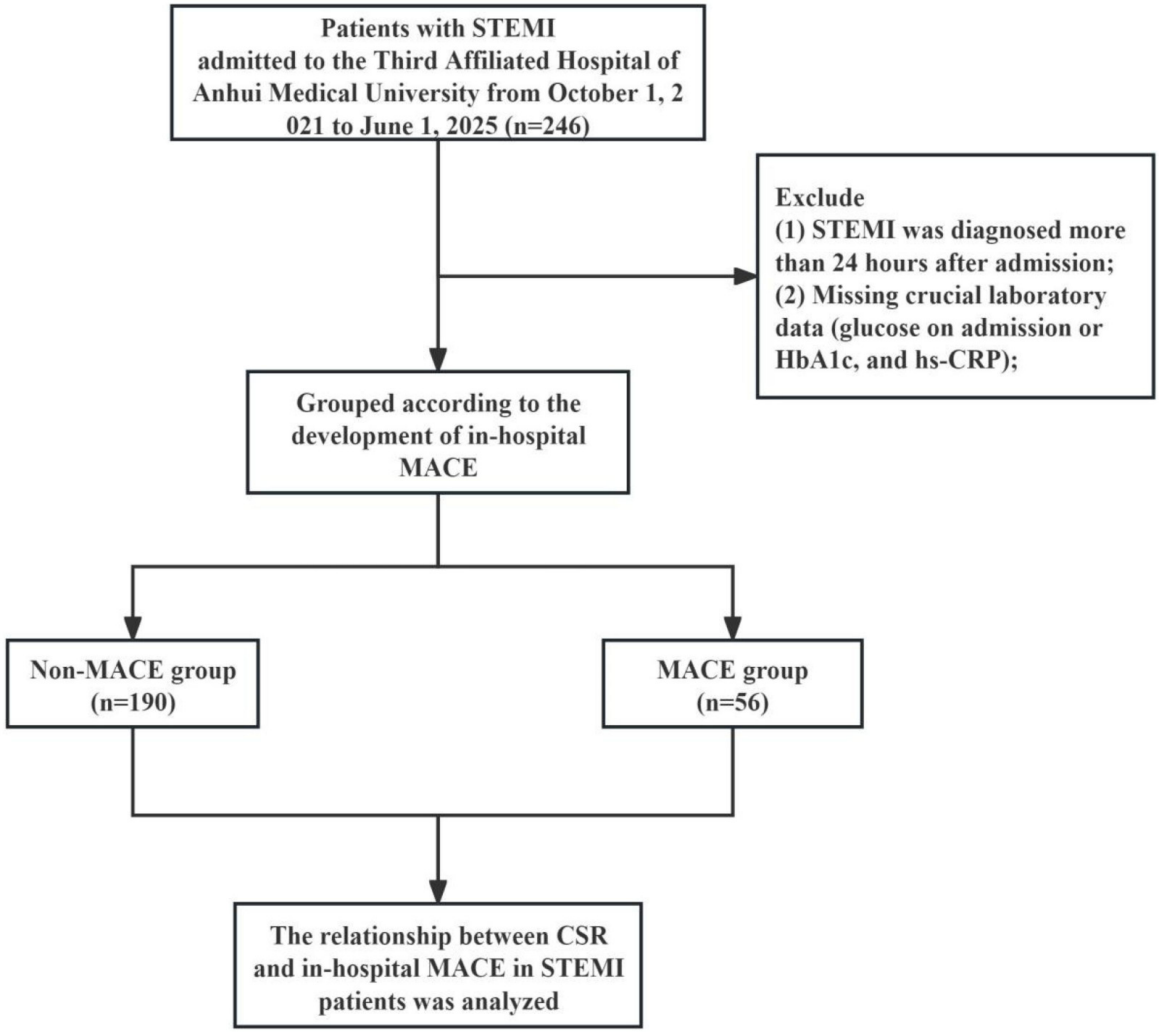
Flow chart of the study.

In this retrospective study, 246 consecutive patients diagnosed with STEMI at our hospital between October 1, 2021, and June 1, 2025, were screened. The diagnosis of STEMI was based on definitive clinical signs of myocardial ischemia and new ischemic changes on the electrocardiogram, specifically new ST-segment elevation in two contiguous leads (≥2 mm in men ≥40 years old in leads V2–V3, ≥2.5 mm in men <40 years old, or ≥1.5 mm in women regardless of age; in other leads: ≥1 mm) (16). Patients were excluded if: (1) STEMI was diagnosed more than 24 h after admission; or (2) key laboratory data were missing [including post-admission blood glucose, glycated hemoglobin (HbA1c), and hs-CRP]. The enrollment process is illustrated in Figure 1.

### Data collection and definitions

The study collected various data from the medical record system, including patient demographic information (such as age, gender, smoking history, alcohol consumption history, hypertension, diabetes, and history of stroke), vital signs data (systolic blood pressure, diastolic blood pressure, and heart rate upon admission), laboratory data within 24 h after admission (neutrophils, monocytes, lymphocytes, hemoglobin, platelets, serum troponin I (cTnI), creatine kinase isoenzyme (CK-MB), myoglobin, N-terminal pro-B-type natriuretic peptide (NT-proBNP), serum creatinine (Scr), blood urea nitrogen (BUN), uric acid (UA), albumin, triglycerides (TG), total cholesterol (TC), high-density lipoprotein cholesterol (HDL-C), low-density lipoprotein cholesterol (LDL-C), blood glucose, glycated hemoglobin (HbA1c), hs-CRP, SHR, and CSR, as well as left ventricular ejection fraction (LVEF), Killip class, GRACE Risk Score, door-to-balloon time (DTB), use of intra-aortic balloon pump (IABP), and number of diseased vessels.

Blood glucose was measured using the glucose oxidase (GOD) method, and glycated hemoglobin (HbA1c) was determined by high-pressure liquid chromatography (HPLC) method. hs-CRP was measured using the immunoturbidimetric method with kits purchased from Wuhan Bairuide Biotechnology Co., Ltd. The normal reference range is 0–10 mg/L.

### Definitions

SHR was defined as the estimated average glucose derived by dividing blood glucose by HbA1c (17). The following formula was used: SHR = blood glucose (mmol/L)/[1.59 × HbA1c (%) – 2.59].

CSR was defined as hs-CRP (mg/L) × SHR/10.

The primary outcome was in-hospital MACE, which, based on previous studies (18, 19), included malignant arrhythmia (VT, lasting ≥30 s or requiring emergency termination due to hemodynamic compromise or VF), acute left heart failure [acute onset or exacerbation of symptoms and signs of heart failure (e.g., dyspnea, orthopnea, pulmonary rales) accompanied by radiographic evidence of pulmonary edema, and requiring intensive intravenous diuretic, vasodilator, or inotropic support], repeat revascularization (unplanned/urgent percutaneous coronary intervention), cardiogenic shock [sustained hypotension (systolic blood pressure <90 mmHg for >30 minutes) with evidence of end-organ hypoperfusion, not due to arrhythmia or hypovolemia, and attributable to cardiac dysfunction], cardiac arrest (cessation of cardiac mechanical activity, as confirmed by the absence of a palpable pulse and unresponsiveness, requiring the initiation of cardiopulmonary resuscitation), and sudden cardiac death (unexpected, non-traumatic death of cardiac cause within 1 h of symptom onset in a patient who was previously clinically stable) (20, 21).

### Statistical analysis

Statistical analyses were performed using SPSS software (version 26.0) and R programming language (version 4.3.0). Statistical significance was defined as a two-tailed *P*-value < 0.05. Continuous variables with normal distribution are expressed as mean ± standard deviation (SD) and compared using t-tests. Non-normally distributed continuous variables are presented as median with interquartile range (IQR) and analyzed with Wilcoxon rank-sum tests. Categorical variables are described using frequencies and percentages, with between-group differences assessed by *χ*^2^ tests. Univariate and multivariate logistic regression analyses were employed to identify risk factors for in-hospital MACE. Prior to performing multivariate logistic regression analysis, we assessed multicollinearity among the independent variables. Variance inflation factor analysis revealed severe multicollinearity between CSR and hs-CRP (both VIFs >10). Given that CSR is a composite parameter integrating information from both hs-CRP and SHR with clearer clinical relevance, only CSR was ultimately included in the final multivariable model to ensure model stability and interpretability of the results. Restricted cubic spline (RCS) curves were used to visualize the relationship between CSR and the incidence of in-hospital MACE. Receiver operating characteristic (ROC) curve analysis was conducted to evaluate the predictive performance of SHR, hs-CRP, CSR, and GRACE Risk Score for in-hospital MACE risk. We further evaluated the incremental value of combining CSR with the GRACE Risk Score by conducting analyses using the Integrated Discrimination Improvement (IDI) index and the Net Reclassification Index (NRI). Additionally, subgroup analysis was performed.

## Results

### Baseline characteristics

A total of 246 patients were ultimately enrolled. Patients were grouped into a MACE group (*n* = 56) and a non-MACE group (*n* = 190) according to the occurrence of in-hospital MACE. In the comparison of baseline characteristics between the in-hospital MACE and non-MACE groups, the following results were observed: In the MACE group, age, history of hypertension, proportion of Killip class 2–4, GRACE Risk Score, door-to-balloon time, use of intra-aortic balloon pump, heart rate on admission, neutrophil count, monocyte count, NT-proBNP, Scr, BUN, blood glucose, hs-CRP, SHR, and CSR were significantly higher than those in the non-MACE group (*P* < 0.05). The MACE group had a significantly lower LVEF than the non-MACE group (*P* < 0.05). No statistically significant differences were found in the other indicators between the two groups (*P* > 0.05) (Table 1).

**Table 1:** Baseline demographic and clinical characteristics of the study population according to in-hospital major adverse cardiovascular events.

Variables	Total (*n* = 246)	Non-MACE group (*n* = 190)	MACE group (*n* = 56)	*P*
Age, years	60.70 ± 13.77	59.69 ± 13.55	64.14 ± 14.07	0.033
Gender, *n* (%)				0.531
Female	54 (21.95)	40 (21.05)	14 (25.00)	
Male	192 (78.05)	150 (78.95)	42 (75.00)	
Smoking, *n* (%)	126 (51.22)	98 (51.58)	28 (50.00)	0.835
Alcohol consumption, *n* (%)	43 (17.48)	30 (15.79)	13 (23.21)	0.199
Hypertension, *n* (%)	130 (52.85)	93 (48.95)	37 (66.07)	0.024
Diabetes, *n* (%)	88 (35.77)	62 (32.63)	26 (46.43)	0.058
Stroke, *n* (%)	34 (13.82)	23 (12.11)	11 (19.64)	0.151
LVEF, %	57.08 ± 8.56	58.51 ± 7.58	52.21 ± 9.87	<0.001
Killip class, *n* (%)				<0.001
1	195 (79.27)	163 (85.79)	32 (57.14)	
2–4	51 (20.73)	27 (14.21)	24 (42.86)	
GRACE Risk Score	119.06 ± 33.23	113.12 ± 29.03	139.21 ± 38.57	<0.001
DTB, min	71.77 ± 19.93	66.91 ± 15.41	88.27 ± 24.39	<0.001
IABP, *n* (%)	15 (6.10)	8 (4.21)	7 (12.50)	0.050
Number of diseased vessels, *n* (%)				0.161
1	70 (28.46)	50 (26.32)	20 (35.71)	
2	72 (29.27)	58 (30.53)	14 (25.00)	
3	104 (42.27)	82 (43.16)	22 (39.29)	
Systolic BP, mmHg	126.75 ± 24.42	127.47 ± 24.19	124.32 ± 25.28	0.398
Diastolic BP, mmHg	76.65 ± 15.37	77.09 ± 15.28	75.14 ± 15.74	0.405
Heart rate	79.13 ± 16.79	77.29 ± 15.38	85.36 ± 19.77	0.006
Neutrophil, 10^9^ /L	8.62 ± 3.74	8.35 ± 3.68	9.52 ± 3.86	0.039
Lymphocyte, 10^9^ /L	1.61 ± 0.88	1.57 ± 0.76	1.76 ± 1.21	0.267
Monocyte, 10^9^ /L	0.60 ± 0.30	0.58 ± 0.28	0.67 ± 0.32	0.041
Hemoglobin, g/L	136.58 ± 18.67	137.56 ± 17.56	133.25 ± 21.89	0.181
Platelet, 10^9^ /L	209.11 ± 61.79	206.20 ± 62.30	218.98 ± 59.50	0.174
cTnI, ng/mL	31.22 (4.46, 50.00)	25.63 (3.61, 50.00)	36.12 (16.57, 50.00)	0.121
CK-MB, U/L	65.00 (21.82, 118.93)	69.97 (20.75, 118.32)	52.85 (24.75, 119.10)	0.907
Myoglobin, ng/mL	476.58 (151.03, 671.18)	465.58 (153.64, 722.44)	511.67 (137.34, 620.80)	0.831
NT-proBNP, pg/mL	751.75 (193.58, 1,559.75)	601.50 (135.75, 1,400.56)	1,553.55 (602.12, 2,625.00)	<0.001
Scr, umol/L	80.63 ± 47.19	77.38 ± 41.16	91.65 ± 62.76	0.047
BUN, mmol/L	6.14 ± 2.80	5.68 ± 2.07	7.69 ± 4.11	<0.001
UA, umol/L	370.48 ± 112.37	363.07 ± 104.44	395.59 ± 133.93	0.057
Albumin, g/L	39.60 ± 3.93	39.77 ± 3.63	38.99 ± 4.79	0.263
TG, mmol/L	1.50 (1.03, 2.35)	1.51 (1.03, 2.41)	1.44 (1.07, 1.96)	0.327
TC, mmol/L	4.38 ± 1.04	4.32 ± 0.99	4.55 ± 1.16	0.154
HDL-C, mmol/L	1.07 ± 0.24	1.05 ± 0.23	1.12 ± 0.28	0.065
LDL-C, mmol/L	2.69 ± 0.92	2.63 ± 0.89	2.87 ± 1.00	0.083
Blood glucose, mmol/L	8.25 ± 3.58	7.96 ± 3.37	9.24 ± 4.09	0.018
HbA1c, %	6.89 ± 1.88	6.83 ± 1.86	7.10 ± 1.94	0.343
hs-CRP, mg/L	4.97 (1.69, 13.70)	4.23 (1.32, 10.82)	9.04 (2.92, 58.93)	<0.001
SHR	1.00 ± 0.28	0.97 ± 0.27	1.07 ± 0.30	0.024
CSR	1.82 ± 4.21	1.02 ± 1.71	4.55 ± 7.70	0.001

LVEF, left ventricular ejection fraction; DTB, door-to-balloon time; IABP, use of intra-aortic balloon pump; BP, blood pressure; cTnI, serum troponin I; CK-MB, creatine kinase isoenzyme; NT-proBNP, N-terminal pro-B-type natriuretic peptide; Scr, serum creatinine; BUN, blood urea nitrogen; UA, uric acid; TG, triglyceride; TC, total cholesterol; HDL-C, high-density lipoprotein cholesterol; LDL-C, low-density lipoproteins cholesterol; HBA1c, glycated hemoglobin; hs-CRP, high-sensitivity C-reactive protein; SHR, stress hyperglycemia ratio; CSR, high-sensitivity C-reactive protein-stress hyperglycemia ratio.

### Association between CSR and in-hospital major adverse cardiovascular events

**Figure 2:**
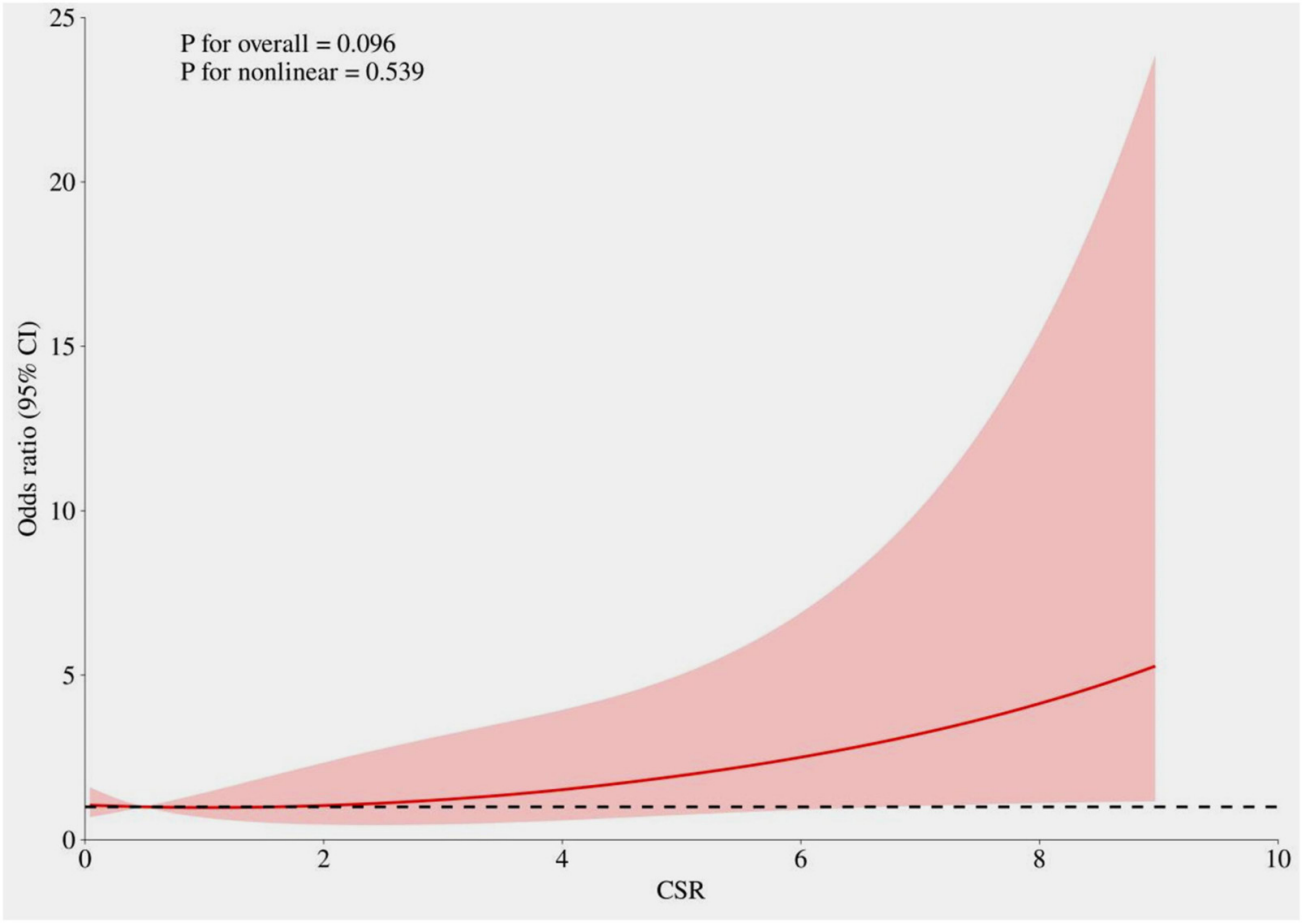
The relationship between CSR and in-hospital major adverse cardiovascular events. The model was adjusted for age, gender, hypertension, diabetes, Killip class, heart rate, neutrophil count, monocyte count, BUN, LVEF, and DTB.

Univariate logistic regression analysis of indicators that showed statistically significant differences between the two groups revealed that age, history of hypertension, LVEF, Killip class, door-to-balloon time, heart rate on admission, neutrophil count, monocyte count, BUN, blood glucose, hs-CRP, SHR, and CSR were risk factors for in-hospital MACE in STEMI patients (*P* < 0.05). Significant variables from the univariate analysis were included in the multivariate regression analysis. Due to concerns regarding multicollinearity among blood glucose, hs-CRP, SHR, and CSR, only CSR was retained in the final model. After adjusting for factors such as gender and diabetes, the multivariate logistic regression analysis demonstrated that history of hypertension, LVEF, Killip class 2–4, door-to-balloon time, and CSR remained independent risk factors for in-hospital MACE in STEMI patients. For each unit increase in CSR, the risk of in-hospital MACE increased by 20.7% (OR = 1.207, 95% CI: 1.024–1.423, *P* = 0.025) (Table 2). As shown in Figure 2, restricted cubic spline analysis indicated that after adjusting for the same covariates included in the multivariate logistic regression model, CSR exhibited a linear positive relationship with the risk of in-hospital MACE (P for non-linearity > 0.05).

**Table 2:** Univariate and multivariate logistic regression analyses of in-hospital major adverse cardiovascular events.

Variables	Univariate logistic regression analysis		Multivariate logistic regression analysis	
	OR (95%CI)	*P*	OR (95%CI)	*P*
Age	1.024 (1.002–1.048)	0.035	1.003 (0.964–1.044)	0.879
Gender (male)	0.800 (0.398–1.608)	0.531	1.099 (0.352–3.425)	0.871
Hypertension	2.031 (1.090–3.783)	0.026	2.596 (1.031–6.535)	0.043
Diabetes	1.789 (0.976–3.281)	0.060	2.314 (0.920–5.824)	0.075
LVEF	0.919 (0.886–0.953)	<0.001	0.927 (0.879–0.978)	0.005
Killip class 2–4	4.528 (2.322–8.29)	<0.001	3.169 (1.147–8.758)	0.026
DTB	1.060 (1.039–1.080)	<0.001	1.080 (1.052–1.108)	<0.001
Heart rate	1.029 (1.010–1.048)	0.002	1.023 (0.999–1.048)	0.058
Neutrophil	1.083 (1.003–1.169)	0.042	1.050 (0.923–1.195)	0.456
Monocyte	2.710 (1.026–7.156)	0.044	1.014 (0.219–4.707)	0.985
NT-proBNP	1.000 (1.000–1.000)	0.105		
Scr	1.005 (0.999–1.011)	0.073		
BUN	1.262 (1.130–1.410)	<0.001	1.081 (0.920–1.271)	0.344
Blood glucose	1.094 (1.013–1.182)	0.022		
hs-CRP	1.026 (1.014–1.037)	<0.001		
SHR	3.016 (1.066–8.534)	0.037		
CSR	1.292 (1.149–1.452)	<0.001	1.207 (1.024–1.423)	0.025

LVEF, left ventricular ejection fraction; DTB, door-to-balloon time; NT-proBNP, N-terminal pro-B-type natriuretic peptide; Scr, serum creatinine; BUN, blood urea nitrogen; hs-CRP, high-sensitivity C-reactive protein; SHR, stress hyperglycemia ratio; CSR, high-sensitivity C-reactive protein-stress hyperglycemia ratio.

### ROC curve analysis

**Figure 3:**
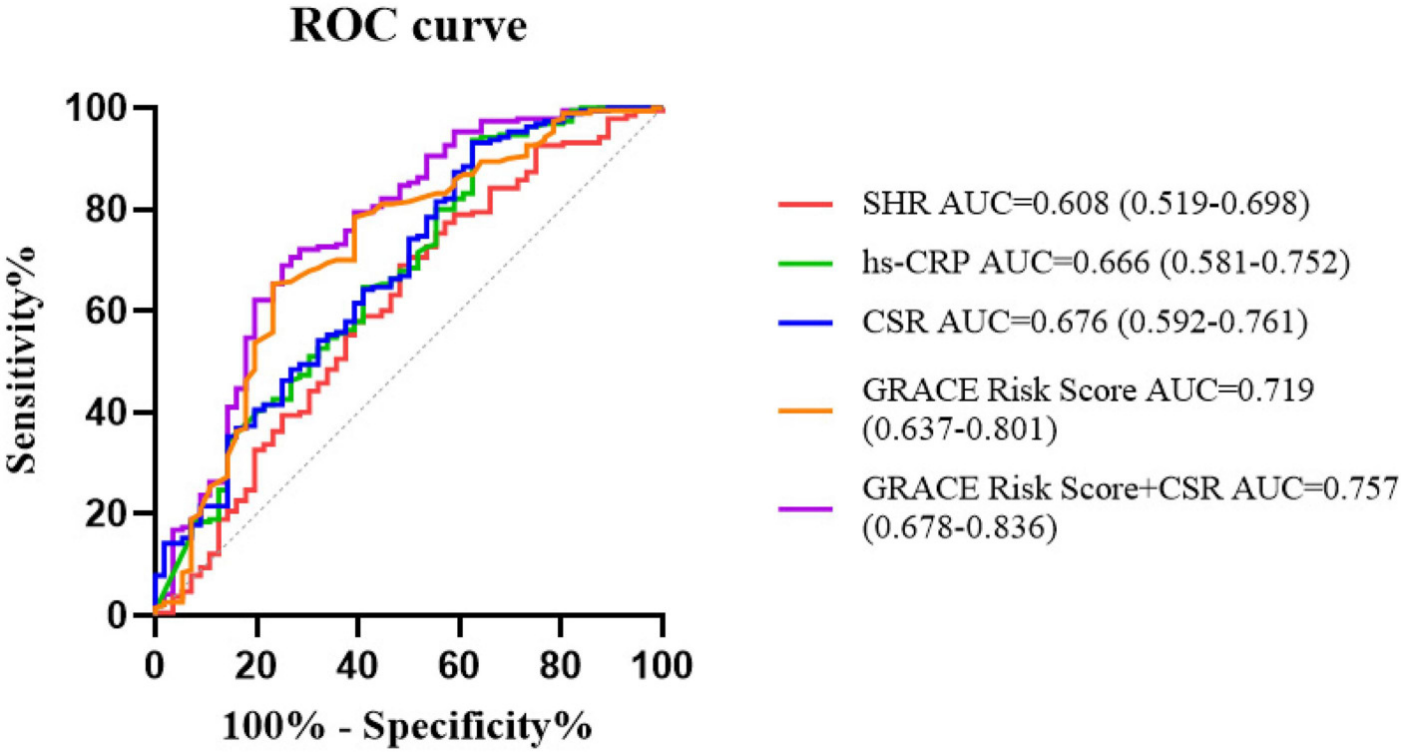
ROC curve.

ROC curve analysis demonstrated that SHR, hs-CRP, CSR, and GRACE Risk Score could independently predict in-hospital MACE in STEMI patients, with area under the curve (AUC) values of 0.608, 0.666, 0.676, and 0.719, respectively (Table 3; Figure 3). Delong test revealed no statistically significant differences in AUC values among these indicators (all *P*-values > 0.05). The combination of the GRACE Risk Score and CSR yields an AUC of 0.757. The results from the NRI and IDI analyses show that the extended model integrating the GRACE Risk Score with CSR leads to a statistically significant improvement in risk reclassification and predictive accuracy compared to the GRACE Risk Score alone (Table 4).

**Table 3:** The predictive value of SHR, hs-CRP, CSR, and GRACE risk score for in-hospital major adverse cardiovascular events.

Index	Cut-off value	AUC (95%CI)	Sensitivity (95%CI)	Specificity (95%CI)	*P*
SHR	1.031	0.608 (0.519–0.698)	0.689 (0.624–0.755)	0.518 (0.387–0.649)	0.014
hs-CRP	35.95, mg/L	0.666 (0.581–0.752)	0.937 (0.902–0.971)	0.375 (0.248–0.502)	<0.001
CSR	3.084	0.676 (0.592–0.761)	0.932 (0.896–0.967)	0.375 (0.248–0.502)	<0.001
GRACE Risk Score	119.5	0.719 (0.637–0.801)	0.653 (0.585–0.720)	0.768 (0.657–0.878)	<0.001
GRACE Risk Score + CSR	–	0.757 (0.678–0.836	0.689 (0.624–0.755)	0.750 (0.637–0.863)	<0.001

SHR, stress hyperglycemia ratio; hs-CRP, high-sensitivity C-reactive protein; CSR, high-sensitivity C-reactive protein-stress hyperglycemia ratio.

**Table 4:** Incremental prognostic value of combined GRACE-CSR model: *Δ*AUC, continuous NRI and IDI.

Comparison	*Δ*AUC	NRI (Continuous)	*P*	IDI	*P*
GRACE Risk Score vs. CSR	0.043 (−0.062 to 0.148)	0.208 (−0.089 to 0.505)	0.169	−0.021 (−0.099 to 0.058)	0.608
GRACE Risk Score + CSR vs. CSR	0.081 (0.003–0.159)	0.514 (0.223–0.804)	<0.001	0.076 (0.033–0.118)	<0.001
GRACE Risk Score + CSR vs. GRACE Risk Score	0.038 (0.008–0.084)	0.299 (0.010–0.588)	0.042	0.096 (0.036–0.156)	0.002

CSR, high-sensitivity C-reactive protein-stress hyperglycemia ratio.

### Subgroup analyses

**Figure 4:**
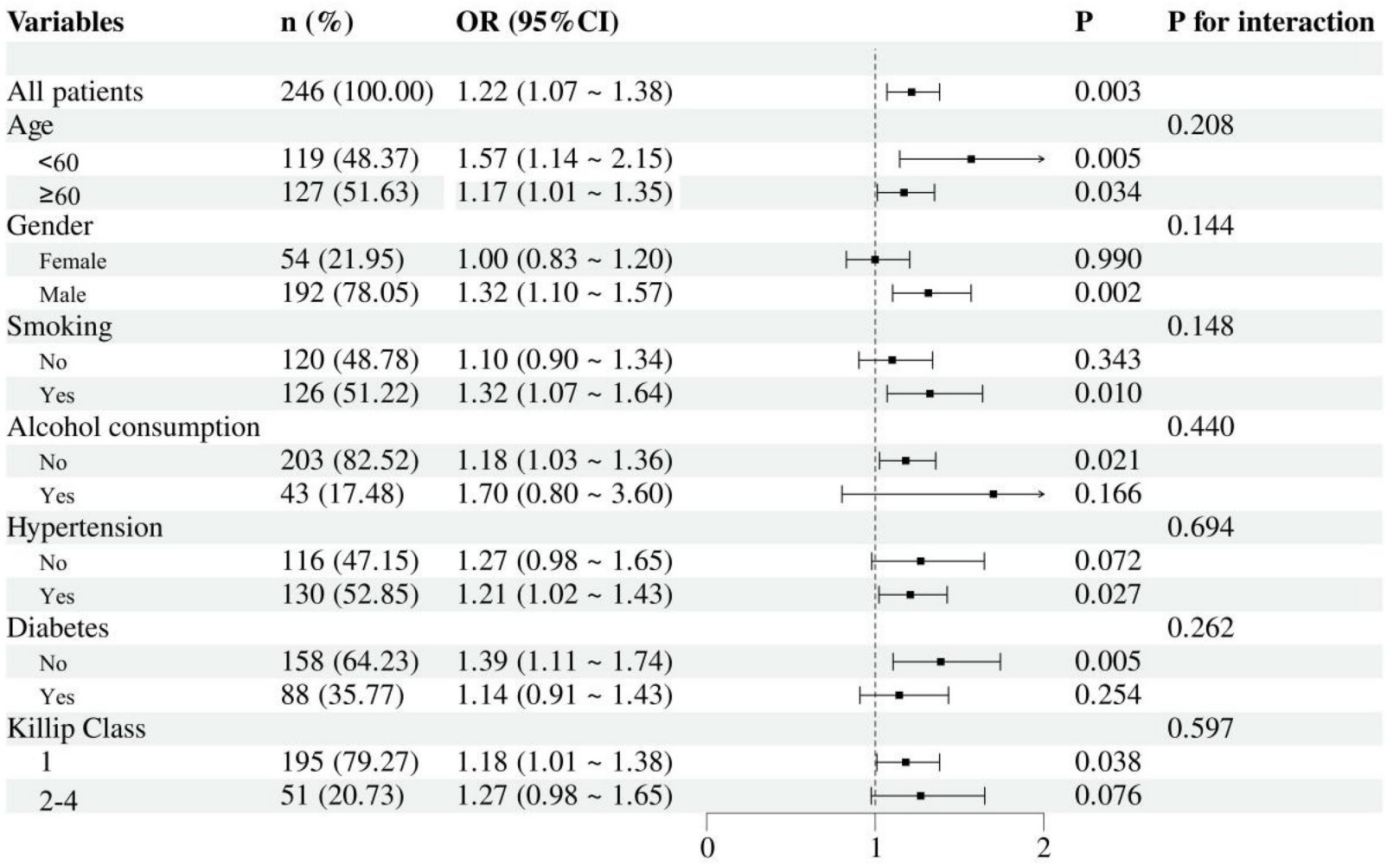
Forest plot of in-hospital MACE risk according to different subgroups. The analysis was adjusted for age, gender, hypertension, diabetes, Killip class, heart rate, neutrophil count, monocyte count, BUN, LVEF, and DTB.

We evaluated the consistency of the association between CSR and in-hospital MACE in STEMI patients through subgroup analysis (Figure 4). The study examined interaction effects across variables including age (<60 years vs. ≥60 years), gender, smoking status, alcohol consumption, comorbidity status (hypertension, and diabetes), and Killip class. The results demonstrated that the association of CSR with in-hospital MACE risk remained consistent across all the aforementioned subgroups (all *P*-values for interaction >0.05).

## Discussion

This study innovatively proposed a novel index, CSR (hs-CRP × SHR/10), which integrates both hs-CRP and SHR, and investigated its predictive value for in-hospital MACE in patients with STEMI. The findings demonstrate that CSR serves as an independent predictor of in-hospital MACE in STEMI patients. When combined with the traditional clinical model GRACE Risk Score, CSR provides a statistically significant improvement in risk reclassification and predictive accuracy compared to using the GRACE Risk Score alone.

The pathophysiological process of acute STEMI involves not only acute thrombotic occlusion but also significant inflammatory activation and metabolic stress. Elevated hs-CRP levels at admission have been confirmed to reflect the intensity of the inflammatory response and are strongly associated with larger infarct size, impaired myocardial reperfusion, and a higher risk of in-hospital MACE (5, 22, 23). Firstly, hs-CRP is a classic nonspecific inflammatory marker produced by the liver in response to interleukin-6 stimulation and plays a pivotal role in the progression of atherosclerosis and plaque destabilization (24). Secondly, as a classic acute-phase reactant, elevated hs-CRP levels directly indicate the intensity of the systemic inflammatory response following myocardial infarction (3). The underlying mechanisms may involve CRP’s participation in promoting plaque instability, activating the complement system, inducing the expression of adhesion molecules on endothelial cells, and inhibiting myocardial repair processes, thereby exacerbating myocardial injury, microvascular dysfunction, and adverse left ventricular remodeling (25, 26). Furthermore, CRP can increase reactive oxygen species levels in AMI patients, enhance the uptake of oxidized LDL, and induce vascular endothelial cell dysfunction and apoptosis (27). Additionally, CRP can stimulate the release of tissue factor from monocytes, endothelial cells, and vascular smooth muscle cells and activate the matrix metalloproteinase system, thereby inducing a prothrombotic state (28).

Patients with acute myocardial infarction often exhibit varying degrees of stress response, leading to elevated blood glucose levels. SHR has emerged as a novel glycemic assessment indicator, and its predictive value in patients with acute myocardial infarction has garnered increasing attention in recent years. Studies have shown that SHR more accurately reflects the body's glycemic status under stress and possesses higher predictive efficacy for both short and long-term prognosis in acute myocardial infarction patients compared to traditional glucose metrics (11, 17, 29). Recent evidence indicates that SHR outperforms admission blood glucose alone in predicting infarct size, microvascular obstruction, and short-term mortality in STEMI patients (9). For example, findings from Xu et al. demonstrated that SHR was significantly associated with the risk of MACE and all-cause mortality in STEMI patients, regardless of diabetic status. Moreover, SHR was more effective than admission blood glucose in predicting 30-day MACE and may enhance the predictive efficiency of the conventional TIMI risk score (10). A study involving 1005 patients with non-ST-segment elevation myocardial infarction (NSTEMI) demonstrated that compared to ABG and HbA1c, SHR showed significantly higher accuracy in predicting type 4a myocardial infarction (30). Liu et al.’s study revealed a significant positive correlation between SHR and both in-hospital mortality and ICU mortality in patients with critical ischemic heart disease. It can improve the predictive accuracy of existing clinical disease scoring systems and guide individualized glucose control (31). Research by Mone et al. indicated that an SHR >1 significantly increased the risk of rehospitalization due to chest pain within one year in patients with ischemia and non-obstructive coronary arteries, suggesting that SHR may serve as an independent risk factor in this population (32). A retrospective analysis of 4,362 patients with coronary artery disease from the COACT registry who underwent PCI, with a median follow-up of 2.5 years, identified SHR as a predictor of major adverse cardiac and cerebrovascular events (MACCE) within 30 days post-PCI (33). It is triggered by the complex neuroendocrine cascade reaction induced by stress hyperglycemia. This involves the activation of the hypothalamic-pituitary-adrenal (HPA) axis, the sympathetic nervous system (SNS), and the renin-angiotensin-aldosterone system (RAAS), leading to elevated levels of insulin-inhibiting hormones, insufficient insulin, and acute insulin resistance (IR) (34–36). SHR may influence prognosis through multiple pathways, including exacerbating endothelial dysfunction, intensifying oxidative stress, promoting the release of pro-inflammatory cytokines, and inducing a hypercoagulable state—all of which collectively increase the risk of heart failure, cardiogenic shock, and death. Studies have shown that CRP levels are significantly higher in patients with stress hyperglycemia than in those with normal glucose levels, and the two markers demonstrate a combined contribution in predicting cardiac dysfunction and adverse events (13, 14).

Most importantly, inflammation and metabolic stress do not exist in isolation but engage in profound pathophysiological crosstalk. On one hand, acute stress-induced hyperglycemia (high SHR) can activate oxidative stress and inflammatory signaling pathways, leading to a further increase in inflammatory factors such as CRP (14). On the other hand, a sustained inflammatory state can also exacerbate insulin resistance, thereby maintaining or even worsening hyperglycemia, forming a vicious cycle that collectively amplifies myocardial damage (14). This study observed that CSR—a novel metric combining CRP and SHR—demonstrated enhanced predictive value when integrated with the traditional clinical model, the GRACE Risk Score. This suggests that simultaneously assessing the inflammatory and metabolic stress axes can provide a more comprehensive perspective for risk stratification.

However, this study has several limitations: (1) This study is a single-center retrospective analysis, and the lack of blinding may introduce potential biases such as measurement bias and recall bias; (2) The timing of CRP measurement (on admission or at peak level) may influence its predictive value; (3) Although multiple confounding factors have been adjusted for, potential unmeasured confounders may still exist. For instance, a study by Wu et al. demonstrated a potential positive association between urinary sodium-to-potassium ratio and myocardial infarction; (4) There was a lack of an external validation cohort to verify the generalizability of the results. Future research directions should include conducting large-scale, multicenter prospective studies to further validate the findings of this study and to explore the clinical application value of CSR.

## Conclusion

In summary, CSR is a reliable biomarker for predicting in-hospital MACE in STEMI patients. It reflects parallel inflammatory and metabolic stress pathways during the disease process. Assessing CSR may help optimize early risk stratification in STEMI patients, provide a basis for individualized treatment, and could ultimately contribute to improving prognosis in high-risk populations.

## Data Availability

The raw data supporting the conclusions of this article will be made available by the authors, without undue reservation.
